# Single-Oocyte Gene Expression Suggests That Curcumin Can Protect the Ovarian Reserve by Regulating the PTEN-AKT-FOXO3a Pathway

**DOI:** 10.3390/ijms22126570

**Published:** 2021-06-18

**Authors:** Yue Lv, Rui-Can Cao, Hong-Bin Liu, Xian-Wei Su, Gang Lu, Jin-Long Ma, Wai-Yee Chan

**Affiliations:** 1 School of Basic Medical Sciences, Shandong University, Jinan 250012, China; lvyue0618@163.com; 2 CUHK-SDU Joint Laboratory on Reproductive Genetics, School of Biomedical Sciences, Faculty of Medicine, The Chinese University of Hong Kong, Hong Kong, China; caoruican@163.com (R.-C.C.); hongbin_sduivf@aliyun.com (H.-B.L.); lugang@cuhk.edu.hk (G.L.); chanwy@cuhk.edu.hk (W.-Y.C.); 3 Center for Reproductive Medicine, Cheeloo College of Medicine, Shandong University, Jinan 250012, China; suxianwei1985@gmail.com; 4 National Research Center for Assisted Reproductive Technology and Reproductive Genetics, Shandong University, Jinan 250012, China

**Keywords:** single cell qPCR, primordial follicle, follicle activation, ovarian reserve, ingenuity pathway analysis, curcumin

## Abstract

A better understanding of the mechanism of primordial follicle activation will help us better understand the causes of premature ovarian insufficiency (POI), and will help us identify new drugs that can be applied to the clinical treatment of infertility. In this study, single oocytes were isolated from primordial and primary follicles, and were used for gene profiling with TaqMan array cards. Bioinformatics analysis was performed on the gene expression data, and Ingenuity Pathway Analysis was used to analyze and predict drugs that affect follicle activation. An ovarian in vitro culture system was used to verify the function of the drug candidates, and we found that curcumin maintains the ovarian reserve. Long-term treatment with 100 mg/kg curcumin improved the ovarian reserve indicators of AMH, FSH, and estradiol in aging mice. Mechanistic studies show that curcumin can affect the translocation of FOXO3, thereby inhibiting the PTEN-AKT-FOXO3a pathway and protecting primordial follicles from overactivation. These results suggest that curcumin is a potential drug for the treatment of POI patients and for fertility preservation.

## 1. Introduction

In the mammalian ovary, primordial follicles are the most basic unit and constitute the ovarian reserves [1]. Under normal circumstances, the majority of primordial follicles remain in a quiescent state and only a few primordial follicles are activated at any particular time. Under some pathological conditions, this balance is disrupted and the activation of primordial follicles is rapidly accelerated leading to diseases such as premature ovarian insufficiency (POI) [2]. In recent years, several genes and pathways have been shown to play important roles in the activation of primordial follicles [3]. Some of these, such as GDF-9, *Grem1/2*, insulin, and BMP4/SMAD signaling [4,5,6,7], promote the activation of primordial follicles, while others, such as *Lhx8*, *Pten*, and Tsc1/mTORC1 signaling, suppress follicle activation [8,9,10]. PTEN-AKT-FOXO3a is a key pathway that regulates the activation of primordial follicles [11,12,13]. The study of primordial follicle activation is significant for clinical research.

POI is a heterogeneous disease that affects about 1% of women of reproductive age [14], and its main etiologies include genetic factors, infections, autoimmune factors, and iatrogenic causes [15]. The lack of ability to have their own genetic offspring remains a significant concern for POI patients, but fortunately about 4–6% of women with POI still have a chance of becoming pregnant with or without hormone therapy [16,17,18]. Using in vitro activation, researchers have found that the PTEN inhibitors bpV and PI3K-activating peptide (740Y-P) can be used to activate the remaining quiescent primordial follicles in the ovaries of POI patients [19], and, in this way, help POI patients successfully give birth to healthy babies [20]. mTOR has also been shown to be involved in follicular activation, and the mTOR agonists phosphatidic acid and propranolol are also identified as potential drug candidates for the treatment of patients with POI [21].

The primordial follicle pool makes up the ovarian reserve, so studying the mechanism of primordial follicle activation can help find ways to maintain the ovarian reserve. The cause of many cases of POI is premature depletion of the ovarian reserve due to overactivation of primordial follicles, which can be the result of genetic factors such as the deletion of the *Pten* gene [10]. In addition, many cases of POI are due to iatrogenic causes, such as when the ovary is exposed to chemotherapeutic agents, and these can lead to the accelerated activation of primordial follicles leading to premature depletion of ovarian reserves, which is usually referred to as the “burnout” effect [22,23,24]. Describing the mechanism of ovarian damage lays the foundation for the identification of potential ovarian reserve protection drugs. Therefore, drugs such as AS101 and melatonin have been shown to reduce the depletion of primordial follicles by inhibiting the PTEN-AKT-FOXO3a pathway [22,25]. In the past decade, many potential drugs have been developed to protect the ovarian reserve. However, more effective and safer drugs are still needed for fertility preservation in the treatment of POI patients.

Here, we studied the mechanism of follicle activation in order to try to identify new drugs to protect the ovarian reserve. We picked 80 oocytes from primordial and primary follicles using the PicoPipet Micro Pick and Place System, and TaqMan array cards (TACs) were used for single-cell qPCR. Ingenuity Pathway Analysis (IPA) was then used to analyze and predict drugs related to follicle activation. We used an in vitro ovarian culture system for drug screening, and we found that curcumin could maintain the ovarian reserve. In vivo experiments showed that long-term treatment of aging mice with curcumin improved their ovarian reserve and showed anti-inflammatory effects. Through further functional experiments, we found that curcumin can reduce the atresia of primordial follicles and can protect against follicle overactivation by inhibiting the PTEN-AKT-FOXO3a pathway.

## 2. Results

### 2.1. Single Oocyte qPCR Using Custom TACs

Firstly, we searched for genes related to the primordial follicle, primary follicle, and POI in the IPA database, and then used the Ovarian Kaleidoscope Database (OKdb) (http://okdb.appliedbioinfo.net/, accessed on 10 September 2018) for gene screening. A total of 91 candidate genes and five internal reference genes were selected to customize the TACs (Table S1). After picking 40 primordial follicles and 40 primary follicle oocytes using the PicoPipet Micro Pick and Place System (Figure S1A), we performed single-cell qPCR using the TACs. After excluding 14 genes with low expression in the qPCR results, we analyzed the remaining 82 genes. The geNorm statistical algorithm was used to evaluate the stability of the 5 reference genes (Figure S1B), and we selected the combination of the top three stable reference genes *Actb*, *B2m*, and *18S* for TaqMan qPCR normalization. We then compared the expression of the 82 candidate genes between the 40 primordial follicle oocytes and the 40 primary follicle oocytes.

All gene expression data were then imported into the Qlucore Omics Explorer software for gene profiling analysis. In the PCA, the two stages of oocytes were clearly divided into two groups (Figure S1C). Figure S1D shows the heatmap of the 82 genes, and hierarchical clustering on the heatmap showed that all oocytes were first divided into two clusters, which was consistent with the PCA results. These results demonstrate that the gene expression profiles were significantly different before and after follicle activation and could be distinguished by single-cell PCR.

### 2.2. Gene Expression in Single Oocytes before and after Follicle Activation

**Figure 1:**
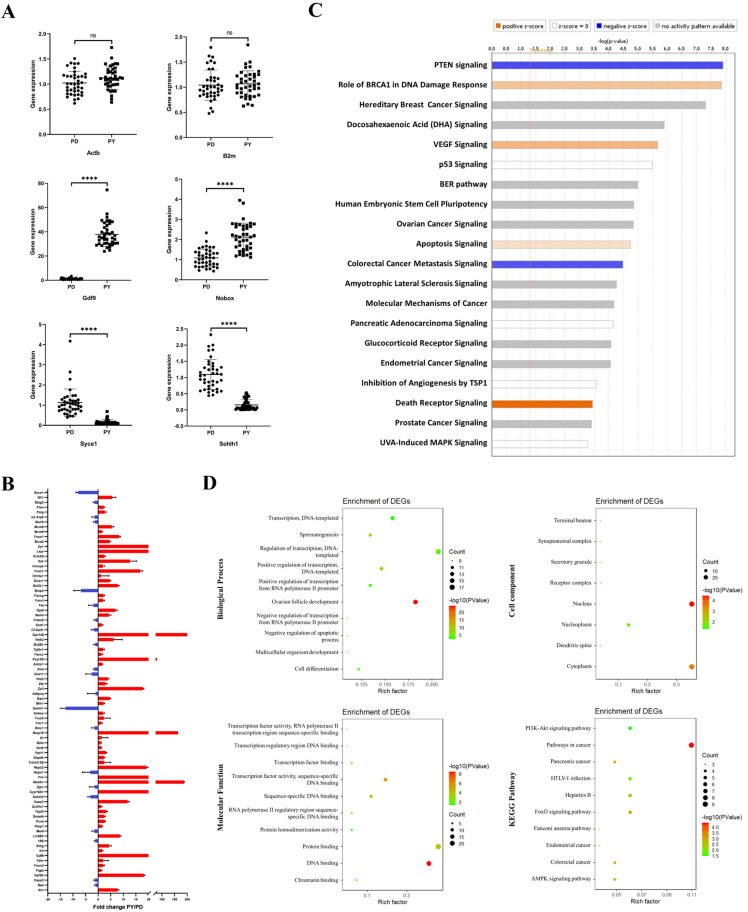
Gene expression in single oocytes. (**A**) The expression of single genes in the 80 oocytes. PD represents the primordial follicle oocytes, and PY represents the primary follicle oocytes. The Y-axis represents fold-changes using 2^−^^△△Ct^, and **** represents *p* < 0.0001. (**B**) The expression of the 82 candidate genes in primary follicles compared with primordial follicles. (**C**) Top 20 canonical pathways predicted by IPA core analysis. The orange columns represent pathways that may promote follicle activation, while blue represents pathways that may inhibit follicle activation. (**D**) GO and KEGG analyses of DEGs.

Next, we observed the expression of individual genes in all single oocytes (Figure 1A). The expression of the reference genes such as *Actb* and *B2m* were not significantly different between the two stages of oocytes, with the fold change being mostly between 0.5 and 1.5. The expression of the oocyte-specific genes *Gdf9* and *Nobox*, which regulate follicle development, significantly increased with the activation of follicles, while the expression of some selected genes, such as *Syce1* and *Sohlh1*, which play important roles in the early stages of meiosis and gametogenesis, decreased. 

Figure 1B shows the expression of all 82 genes, and it is clear that the expression of most selected candidate genes increased during follicle activation. Subsequently, we input all of the expression data into IPA for biological information analysis, including canonical pathway analysis, upstream regulator prediction, etc. Figure 1C shows that the predicted top 20 canonical pathways, and the PTEN pathway, which plays a role in maintaining follicle quiescence, has the highest score. We then performed gene ontology (GO) analysis of 51 differentially expressed genes (DEGs) that showed more than 2-fold differences between the two groups (Figure 1D). Using GO analysis, we found that the putative target genes are involved in biological processes such as DNA-dependent transcription and ovarian follicle development. GO analysis also showed that these putative targets are involved in several molecular functions mainly focused on protein binding and DNA binding. In addition, cell component analysis showed that the proteins encoded by these putative target genes mainly localize to the nucleus and cytoplasm. The KEGG (Kyoto Encyclopedia of Genes and Genomes) analysis showed that in addition to cancer pathways, pathways related to follicle activation, such as the PI3K-AKT pathway and FOXO pathway, were also on the list. 

### 2.3. Curcumin Reduces the Atresia of Primordial Follicles in order to Maintain Ovarian Reserve

**Figure 2:**
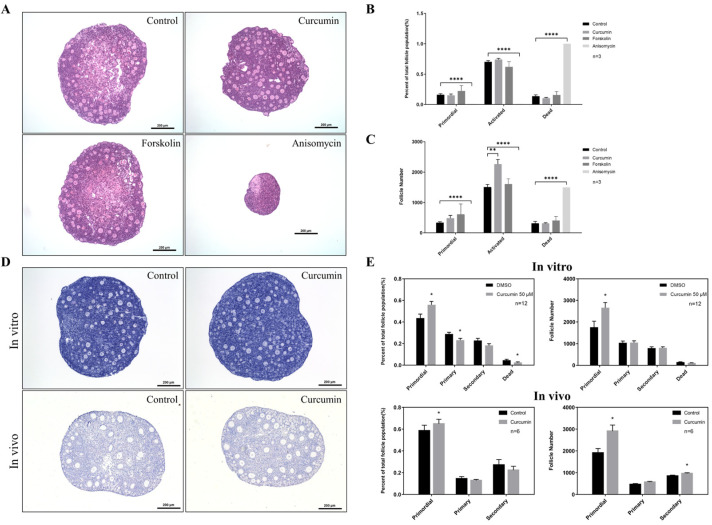
Curcumin reduces the degradation of primordial follicles to maintain the ovarian reserve. (**A**) The ovarian in vitro culture system was used for drug screening, and ovaries from 3-to-4-day-old mice were cultured for 12 days. (**B,C**) Follicle counting after treatment with different drugs. B shows the distribution of different stages of follicles, while C shows the number of follicles. (**D**) Histological changes in the ovary after curcumin treatment. In vitro experiments used P3 ovaries cultured for 7 days, and in vivo experiments used P5 mice with continuous ip injection of 100 mg/kg curcumin for 7 days. (**E**) Follicle counting after treatment with curcumin in vivo and in vitro. FC: follicle. * *p*-value < 0.05, ** *p*-value < 0.01, **** *p*-value < 0.0001 compared to the controls.

As mentioned above, we predicted the upstream regulators of selected genes using IPA (Table S3). Then, we selected some chemical compounds from this list (Table S3) and used the ovarian in vitro culture system for drug screening. The purpose was to identify drugs that have an effect on the follicle activation process. Figure 2A shows the results of three examples, namely curcumin, forskolin, and anisomycin. The ovaries of 3-to-4-day-old neonatal mice, which mainly contain primordial follicles, were first cultured with the drug-containing medium for 48 h. The medium was then changed to the normal ovarian culture medium. After 12 days of culture, we found that the oocytes in the ovaries cultured with curcumin were large and round, and there was little necrosis in the center of the ovary. In addition, we also observed no significant difference between the ovaries treated with forskolin and the control group. However, anisomycin was obviously toxic to the ovarian cells and caused the death of all follicles (Figure 2A). The follicle counts showed that the number of activated follicles increased significantly after curcumin treatment, but the number and percentage of primordial follicles did not decrease (Figure 2B,C). Therefore, we speculated that curcumin might inhibit follicle atresia under in vitro culture conditions.

In addition, we conducted in vivo experiments by intraperitoneally (ip) injecting curcumin into 5-day-old mice for 7 consecutive days and then comparing the histology of the ovaries treated in vivo with the ovaries after 7 days of in vitro culture (Figure 2D). The proportion and number of primordial follicles in ovaries cultured in vitro were both significantly higher than those in the control group, while the proportion of primary follicles was lower (Figure 2E). In addition, the proportion of apoptotic follicles also decreased. The results of the in vivo experiments were consistent with the in vitro experiments, and both the proportion and number of primordial follicles increased significantly after curcumin treatment. The results indicated that short-term treatment with curcumin can reduce the atresia of primordial follicles and help maintain the ovarian reserve.

### 2.4. Curcumin May Have a Protective Effect on the Fertility of Aging Mice

**Figure 3:**
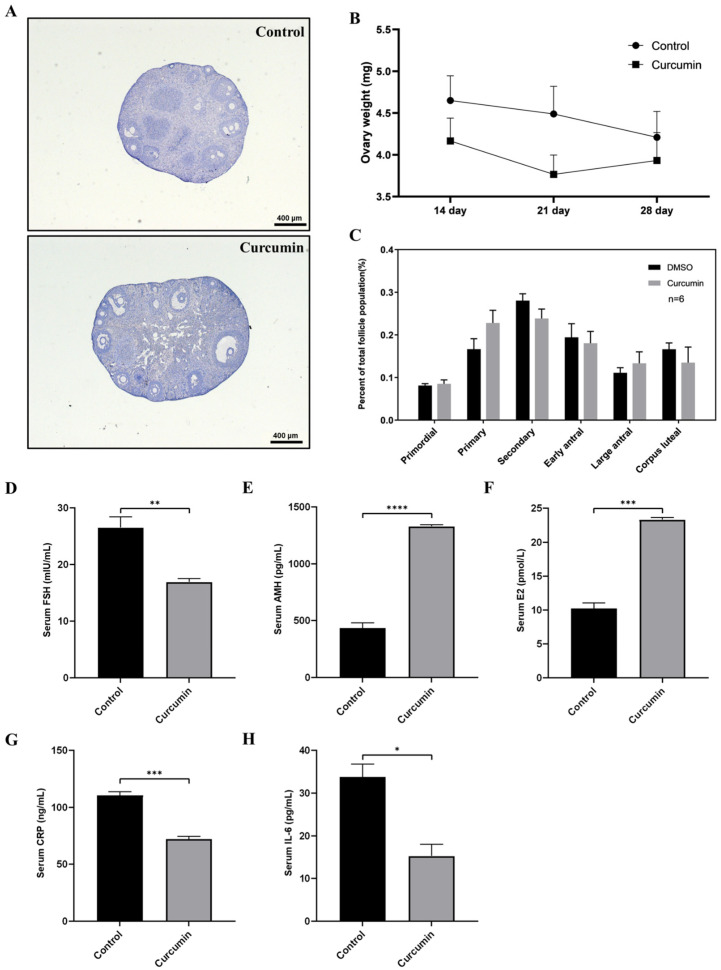
The effect of curcumin on aged mice. (**A**) Histology of ovaries after ip injection of curcumin in aged mice. (**B**) The weight of the ovaries of aged mice at different time points. (**C**) The follicle counts in aged mice. (**D**–**H**) Expression levels of reproductive hormones and inflammatory factors in the serum of aged mice. * *p*-value < 0.05, ** *p*-value < 0.01, *** *p*-value < 0.001, **** *p*-value < 0.0001 compared to the controls.

To investigate the effect of long-term curcumin treatment on the fertility of aging mice, we injected curcumin ip at 100 mg/kg into 7-month-old mice for 28 consecutive days. After treatment, we observed the weight and histological changes of the ovaries and found that there were no significant changes between the curcumin group and the control group (Figure 3A,B). In addition, we found no significant differences in the distribution of follicles in each period (Figure 3C). Therefore, we concluded that curcumin has no significant effect on the follicle development of aging mice, and that long-term use of curcumin has no significant adverse effects on mice.

Subsequently, we used ELISA to measure serum hormone levels, including FSH, AMH, and E2 of these aging mice. Surprisingly, the serum FSH level of the aged mice in the curcumin treatment group was significantly decreased, while the levels of AMH and E2 were significantly increased (Figure 3D–F). As a key indicator of ovarian reserve [26], the increase in AMH indicates that curcumin might have a protective effect on the ovarian reserve. In addition, FSH and E2 are often used as clinical indicators of ovarian reserve, and their changes further suggest that curcumin can regulate the serum hormone levels of aging mice and that it has a protective effect on the ovarian reserve [27]. To investigate the anti-inflammatory effects of curcumin, we also measured the inflammatory factors CRP and IL-6 in serum. We found that after curcumin treatment, the level of CRP and IL-6 were significantly reduced (Figure 3G,H), indicating that curcumin also has an anti-inflammatory effect in aged mice.

### 2.5. Curcumin Protects against Follicle Overactivation by Inhibiting the PTEN-AKT-FOXO3a Pathway

Next, we studied the molecular mechanism for how curcumin maintains the primordial follicles in a quiescent state. The results in Figure 1C showed that the Pten signaling pathway is the most relevant pathway related to follicle activation, and the KEGG analysis of DEGs also suggested that the PTEN-AKT-FOXO3a pathway might play an important role in the process of follicle activation (Figure 1D). Table S3 shows that PTEN is a target molecule of curcumin, and therefore we speculated that curcumin might also play a role through the PTEN-AKT-FOXO3a pathway. To investigate the effect of curcumin on the PTEN-AKT-FOXO3a pathway, we added the PTEN inhibitor bpV as a positive control. Curcumin, bpV, and the combination of the two drugs were used to treat cultured ovaries, and we analyzed the gene expression, protein expression, and protein localization in the treated ovaries.

**Figure 4:**
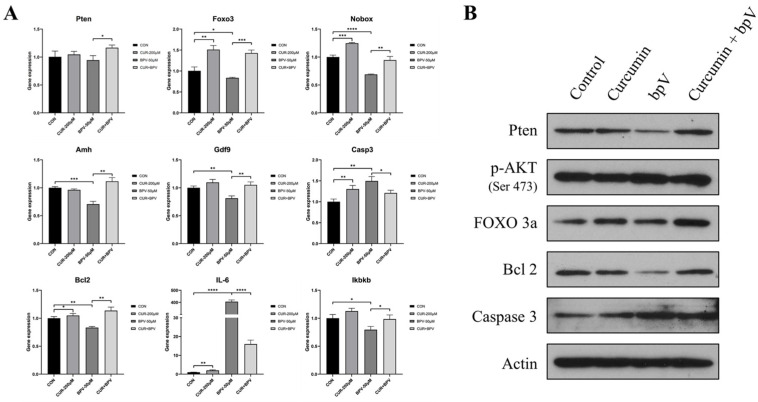
Curcumin protects against follicle overactivation by inhibiting the PTEN-AKT-FOXO3a pathway. (**A**) Gene expression of P3 ovaries cultured with curcumin (CUR) and/or bpV for 2 days. (**B**) Western blot of proteins from ovaries cultured with curcumin and/or bpV for 12 h. * *p*-value < 0.05, ** *p*-value < 0.01, *** *p*-value < 0.001, **** *p*-value < 0.0001 compared to the corresponding groups.

To investigate the effect of curcumin on cultured ovaries, we observed changes in the expression of some genes that are specifically expressed in oocytes, including *Nobox*, *Amh*, and *Gdf9* (Figure 4A). The results show that, after treatment with bpV, the expression of *Amh* in the cultured ovaries significantly decreased. However, when bpV and curcumin were used at the same time, *Amh* expression was similar to controls. Combined with ELISA data of serum AMH, these results demonstrate the protective effect of curcumin on ovarian reserves (Figure 3E). We also observed the effect of curcumin on apoptosis-related factors and found that co-treatment of ovaries with curcumin and bpV reduced the expression of CASP3 and increased the expression of BCL2. The results also show that the inflammatory factor Il-6 was significantly increased after bpV treatment, and curcumin could inhibit this increase. In addition, curcumin also regulated the expression changes of *Ikbkb* (Figure 4A). Therefore, we speculate that curcumin may exert its anti-inflammatory effect by influencing IL-6 activity through the regulation of Ikbkb.

**Figure 5:**
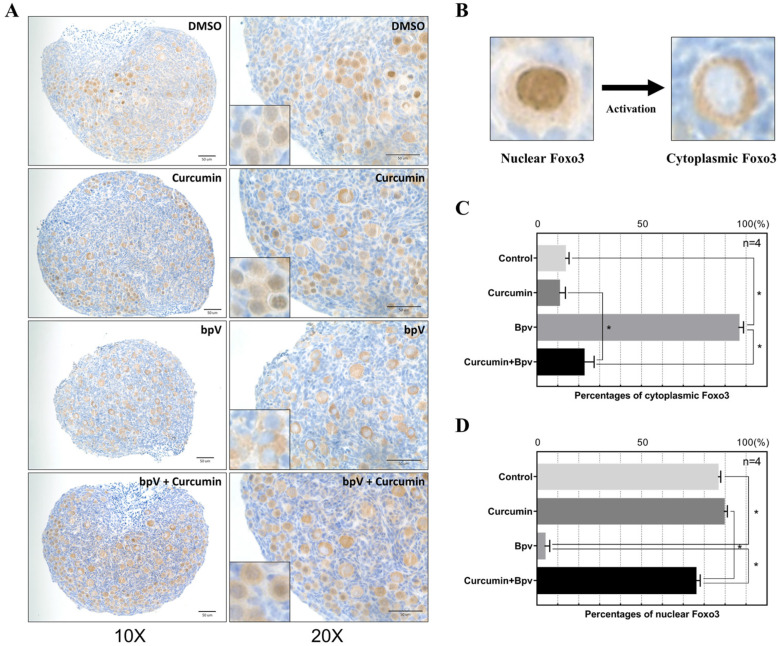
Curcumin inhibits follicle activation by reducing the translocation of FOXO3. (**A**) The ovaries of different treatments were collected for IHC staining with antibodies against FOXO3. The enlarged picture is shown at the bottom left. (**B**) Schematic diagram of nuclear and cytoplasmic FOXO3. (**C,D**) The percentage of cytoplasmic (**C**) and nuclear (**D**) FOXO3 from P3 ovaries treated with curcumin and/or bpV, * indicates *p*-value < 0.05.

The mRNA and protein expression of PTEN did not change significantly after drug treatment (Figure 4A), but Western blot showed that bpV treatment significantly inhibited PTEN expression (Figure 4B). When both drugs were used to treat the ovary, the inhibitory effect of PTEN was reversed. In addition, we found that *Foxo3* gene and protein expression both changed significantly after drug treatment. Curcumin could significantly increase the gene expression of *Foxo3*, and Western blot also showed that the total amount of FOXO3 increased significantly after treating with curcumin, while *p*-FOXO3 decreased (Figure 4B). Therefore, we speculate that curcumin could inhibit the translocation of *p*-FOXO3. After the addition of bpV, the total FOXO3 protein level decreased because bpV promotes AKT phosphorylation, thereby increasing the phosphorylation of FOXO3 that then activates the primordial follicles. Studies have shown that when FOXO3 is in the nucleus of the oocyte, it will maintain the follicles in a quiescent state. However, when FOXO3 is phosphorylated, it translocates out of the nucleus to promote follicle activation [28]. To confirm this, we performed IHC staining of FOXO3 (Figure 5A). After a short culture for 6 h with the PTEN inhibitor bpV, FOXO3 translocation was observed in almost all primordial follicles. However, after adding curcumin and bpV at the same time, the percentage of FOXO3 in the nucleus was not significantly different from controls (Figure 5C,D). In addition, curcumin could reverse the overactivation of follicles caused by the activation of the PTEN-AKT-FOXO3a pathway. Therefore, we concluded that curcumin can maintain the quiescence state of the primordial follicle, and can protect the primordial follicle from being overactivated by regulating the PTEN-AKT-FOXO3a pathway. 

## 3. Discussion

Describing the mechanism of primordial follicle activation lays the foundation for the identification of potential drugs that can activate follicles or protect the ovarian reserve, thus providing the possibility for the treatment and prevention of POI. In the present study, we used single-cell qPCR to describe the gene expression profiles during follicular activation, and found that curcumin has a protective effect on the ovarian reserve by regulating the PTEN-AKT-FOXO3a pathway.

The ovary contains many cell types, and the development of follicles is not synchronized [29,30]. Therefore, we used TACs to analyze the gene expression of a single oocyte before and after activation in order to avoid the influence of other cells. TACs are low-throughput arrays that have the advantages of accuracy and flexibility, and require only a small amount of cDNA, and they are widely used in clinical pathogen diagnosis and in studies of candidate genes/miRNAs [31,32,33]. This technique allowed us to more accurately analyze the expression of candidate genes that are activated in follicles compared to RNA-sequencing or other methods. In order to identify new drugs that can regulate the activation of follicles, we used IPA for bioinformatics analysis of gene expression profiles. IPA is a commonly used tool for signal pathway research and drug screening [34], and core analysis in IPA is often used to predict upstream regulatory factors and drugs in various research fields [35,36,37]. For example, by using IPA to analyze the differences in the transcriptomes of follicular development in vitro and in vivo, a previous study predicted follicular development-related signaling pathways and drugs and found that adding an estrogen-receptor antagonist to cultured ovarian tissue (ICI 182,780) can overcome hypoplastic follicular development in vitro [37]. In the present study, the results of the IPA canonical pathway analysis show that the PTEN pathway scored the highest and was predicted to play a role in maintaining follicle quiescence (Figure 1C). Combined with the GO analysis of DEGs (Figure 1D), these results further confirm with single-cell data that the PTEN-AKT-FOXO3a signaling pathway is the key pathway for follicular activation. Next, we predicted the upstream factors that might be associated with follicle activation (Table S3) and selected chemical drugs with the highest z-scores and verified their effects in the ovarian in vitro culture system, which is commonly used to study primordial follicle activation [21,28,38]. Among these drugs, curcumin was found to have a protective effect on the ovarian reserve. 

Curcumin, also called diferuloylmethane, is the main natural polyphenol found in the rhizome of *Curcuma longa* (turmeric) [39], which is extensively used as a spice, as a food coloring, and as a traditional herbal medicine [40]. Curcumin can be safely used in humans, and curcuminoids have been approved by the US Food and Drug Administration as “Generally Recognized as Safe” [41]. In this study, our results in mice also demonstrate the safety of curcumin, and long-term treatment (28 days) with curcumin had no significant effect on the body weight of mice (Figure 3B). Curcumin has been shown to have many health benefits through its antioxidant, anti-inflammatory, and anti-cancer activities [42,43,44,45]. Through these effects, curcumin has potential for use against diabetes, allergies, arthritis, Alzheimer’s disease, and other chronic illnesses [46]. Our study also shows that long-term curcumin treatment leads to changes in AMH and FSH, which are indicators of ovarian reserve, thus indicating its potential for maintaining fertility. Not only that, the reduction of serum inflammatory factors IL-6 and CRP indicates that curcumin also exerts an anti-inflammatory effect. 

Studies have shown that curcumin has a protective effect on the female reproductive system [47,48], and it has been reported that dietary turmeric can improve the reproduction, ovarian function, and growth of rabbits and can stimulate the growth of follicles at all stages of folliculogenesis [49]. In addition, it is reported that curcumin can protect the ovaries from damage. For example, curcumin can protect the ovaries and reduce the damage caused by cyclophosphamide [50]. It can also protect against D-galactose-induced POI [48]. In addition, curcumin also has a protective effect on rat ovarian ischemia-reperfusion injury [51,52]. Most of these studies show that the antioxidant effect of curcumin plays an important role in ovarian protection, but the mechanism of how curcumin protects the ovarian reserve has not been studied in detail. In this study, we found that curcumin can significantly increase the expression of follicle activation and maintenance factors such as the *Foxo3* and *Nobox* genes. In addition, curcumin can also inhibit the follicle activation effect of bpV by reducing the translocation of FOXO3. Therefore, we conclude that curcumin can protect primordial follicles from overactivation by inhibiting the PTEN-AKT-FOXO3a pathway.

In conclusion, through single-oocyte qPCR and ovarian in vivo and vitro experiments we show that as a food additive and herbal medicine curcumin has a systemic effect on mice. We found that it can affect the female reproductive system through anti-inflammatory and endocrine regulation, and mechanistic studies show that curcumin maintains the stability of the ovarian reserve by regulating the PTEN-AKT-FOXO3a pathway. Taken together, our results suggest that curcumin can be used to prevent ovarian damage caused by chemotherapy and can also be used as a potential drug for the treatment of patients with iatrogenic POI.

## 4. Materials and Methods

### 4.1. Animals

Female C57BL/6 mice aged 3–5 days old, 8–10 days old, and 6–8 months old were used in this experiment. The 3-to-4-day-old and 8-to-10-day-old mice were provided by the Laboratory Animal Services Centre of the Chinese University of Hong Kong, and the 30 6-to-8-month-old mice were provided by the Shandong University Laboratory Animal Center. 

### 4.2. Custom TaqMan Array Cards

A total of 91 candidate genes for follicle activation and 5 endogenous reference genes were selected from the IPA software and OKdb [53] to customize the TACs (SKU 4342259, ThermoFisher Scientific, Rockford, IL, USA). We searched for the assay IDs of these genes to design a TAC using the online tool on the Thermo Fisher Scientific website (https://www.thermofisher.cn/order/custom-array/configure, accessed on 16 October 2018) (Table S1). We chose Format 96a in which 96 genes are repeated four times in one 384-well card. A total of 20 custom TACs were ordered.

### 4.3. Single Oocyte Isolation

Female C57BL/6 mice aged 8–10 days old were sacrificed and their ovaries were removed under a dissecting microscope. A 28-gauge needle was used to separate and remove the surrounding fat and other tissues, and the ovaries were transferred to a digestion medium consisting of aMEM supplemented with 5 mg/mL Liberase (Sigma-Aldrich, St. Louis, MO, USA), 10 mg/mL collagenase type IV (Sigma-Aldrich), and 5% DNase I (STEMCELL Technologies, Vancouver, BC, Canada) and incubated at 37 °C for 45 min. The digestion was stopped by adding culture medium (aMEM (ThermoFisher Scientific) supplemented with 10 mIU/mL follicle stimulating hormone (FSH, Sigma-Aldrich), 3 mg/mL bovine serum albumin (BSA, Sigma-Aldrich), 1 mg/mL fetuin (Sigma-Aldrich), 1% insulin-transferrin-selenium (ITS-G, ThermoFisher Scientific) and 1% penicillin/streptomycin (ThermoFisher Scientific)). The medium was filtered twice with a 70 μm cell strainer and transferred to a hanging 8 μm pore size SPLInsert Hanging (SPL Life Sciences, Gyeonggi-do, Korea). The solution was then washed three times to filter out the granulosa cells and other ovarian cells. The cells remaining in the SPL insert, which mainly consisted of oocytes, were resuspended in 1 mL culture medium. Single oocytes were then transferred to 200 μL tubes using the PicoPipet Micro Pick and Place system (Nepagene, Chiba, Japanese) for subsequent cell lysis.

### 4.4. Single Oocyte qPCR and Pooled Cell qPCR

The single oocytes were prepared using the Single Cell-to-Ct Kit (ThermoFisher Scientific) following the manufacturer’s instructions and loaded onto a TAC for qPCR. The reverse transcription and preamplification steps were conducted in a clean PCR workstation. After pre-amplification, the cDNA was diluted with 1× TE buffer at 1:20. The TACs were run in a QuantStudio 7 Flex Real-Time PCR machine (ThermoFisher Scientific), which contains a TaqMan array block. The RNA from pooled cells was reverse transcribed using a SuperScript VILO cDNA Synthesis Kit (ThermoFisher Scientific), and qPCR was performed with the Power Up SYBR Green Master Mix (ThermoFisher Scientific) following the manufacturer’s instructions. The qPCR primer sequences are shown in Table S2.

### 4.5. In Vitro Ovary Culture and Follicle Counting

The 3-to-4-day-old mouse ovaries were dissected and placed on the surface of a 12 mm cell culture plate insert with 0.4 μm pore size (Sigma-Aldrich). The culture inserts were placed in 6-well plates containing 1.6 mL culture medium (DMEM/F12 supplemented with 1 mg/mL BSA, 1 mg/mL Ablumax II (ThermoFisher Scientific), 5% ITS-G, 100 µM L-ascorbic acid (ThermoFisher Scientific), and 1% penicillin/streptomycin) in each well, and 4–6 ovaries were cultured on each culture insert. Different drugs including curcumin (Cat No. S1848, Selleckchem, Houston, TX, USA), forskolin (Cat No. S2449, Selleckchem), anisomycin (Cat No. S7409, Selleckchem) and Bpv (Cat No. S8651, Selleckchem) were added to the medium during the ovarian culture. The culture medium was changed every other day.

The cultured ovaries were embedded in paraffin and sectioned at a thickness of 4 μm then stained with HE. Every fifth section from each ovary was used for counting. Only follicles with clearly stained oocyte nuclei were counted in order to prevent the recounting of the same follicle. Follicles with one oocyte surrounded by a single layer of flattened granulosa cells were scored as primordial follicles. Often, follicles contained a single granulosa layer that consisted of both flattened and cubical granulosa cells, and these were also scored as primordial follicles. Follicles with an oocyte surrounded by one layer of cubical granulosa cells were considered primary follicles, and follicles with an oocyte surrounded by two or more layers of granulosa cells were considered secondary follicles. In this study the primary, secondary, and other advanced stages of follicle were collectively called developing follicles. Finally, to estimate the total number of follicles per ovary, all follicle numbers presented in the marked sections were multiplied by five.

### 4.6. Western Blot

Total protein was extracted using Neuronal Protein Extraction Reagent (ThermoFisher Scientific), and proteins were separated by electrophoresis on a 12% polyacrylamide gel and transferred to a PVDF membrane (Millipore, Billerica, MA, USA). The membranes were incubated overnight with the primary antibodies at 4 °C with optimal dilution following the manufacturer’s recommendations, and then incubated with the appropriate secondary antibody at a 1:2000 dilution for 1 h at room temperature. The FOXO3 (Cat No. 2497, Cell Signaling Technology, Danvers, MA, USA), *p*-FOXO3 (Thr32) (Cat No. 9464S, Cell Signaling Technology), *p*-AKT (Ser473) (Cat No. 4060, Cell Signaling Technology), BCL2 (Cat No. 7382, Santa Cruz Biotechnology, Santa Cruz, CA, USA), CASP3 (Cat No. 7184, Santa Cruz Biotechnology), PTEN (Cat No. 9188, Cell Signaling Technology), and ACTIN β (Cat No. 4967, Cell Signaling Technology) primary antibodies were used in the Western blot experiments. The membranes were then incubated with horseradish peroxidase-conjugated secondary antibodies (1:2000; Cell Signaling Technology) for 1 h at room temperature. The specific signals were detected with enhanced chemiluminescence (Western Bright ECL HRP substrate; Advansta, Menlo Park, CA, USA) and Lucent Blue X-ray film (Advansta).

### 4.7. Immunohistochemistry (IHC)

The cultured ovaries were obtained from 3-day-old mice and collected for paraffin sectioning at a thickness of 4 μm. Antigen retrieval was performed by boiling the sections in citrate buffer for 25 min and slowly cooling, and endogenous peroxidase activity was blocked using hydrogen peroxide for 10 min. Immunohistochemical analysis was performed using a rabbit-specific IHC kit (Abcam, Cambridge, MA, USA). The sections were incubated with FOXO3 primary antibodies with slow shaking at 4 °C overnight and then stained with DAB.

### 4.8. Enzyme-Linked Immunosorbent Assay (ELISA)

ELISA kits for mouse FSH (A05021-2), estradiol (E2) (A05182-2), anti-Müllerian hormone (AMH) (N04308-2), c-reactive protein (CRP) (N03844-2), and interleukin-6 (IL-6) (N03981-2) were all from Shanghai Jining Shiye Co., Ltd., Jining, China, and were used according to the manufacturer’s instruction.

### 4.9. Statistical Analyses

The expression stability of the 5 reference genes was analyzed using the R-based algorithm geNorm as previously described [54]. Principal component analysis (PCA) and heatmaps were generated using Qlucore Omics Explorer [55]. Core analysis was performed with the IPA software [56], and canonical pathways and upstream regulators were predicted. Independent samples t-tests were performed using GraphPad Prism 8 (GraphPad Software, Inc., La Jolla, CA, USA), and a value of *p* < 0.05 was regarded as statistically significant. Annotations for GO and KEGG analyses were performed with the online tool DAVID 6.8 (https://david.ncifcrf.gov/, accessed on 9 June 2020), and the data visualization was performed in R.

## Data Availability

The data that support the findings of this study are available from the corresponding author upon reasonable request.

## Supplementary Materials

The following are available online at https://www.mdpi.com/article/10.3390/ijms22126570/s1.
