# Supplementary material for: Single-Oocyte Gene Expression Suggests That Curcumin Can Protect the Ovarian Reserve by Regulating the PTEN-AKT-FOXO3a Pathway
Source: Int J Mol Sci. 2021 Jun 18;22(12):6570. doi: 10.3390/ijms22126570 (PMC8235657; doi:10.3390/ijms22126570)
Supplement: Supplementary file 1 [file ijms-22-06570-s001.zip › ijms-1252127-supplementary/Supplementary.pdf]

Supplementary Figure S1. Single oocyte isolation and candidate gene expression profiling. (**A**) Isolated single oocytes. The diameter of the glass mi-cropipette in the left figure is 30 μm, and the diameter of the glass micropipette in the right figure is 50 μm. (**B**) The mean M-value of the 5 reference genes using the geNorm algorithm. (**C**) PCA of 80 single oocytes. (**D**) Heatmap and hierarchical clustering analysis of 80 oocytes. PD: Primordial follicle oocytes, PY: Primary follicle oocytes.

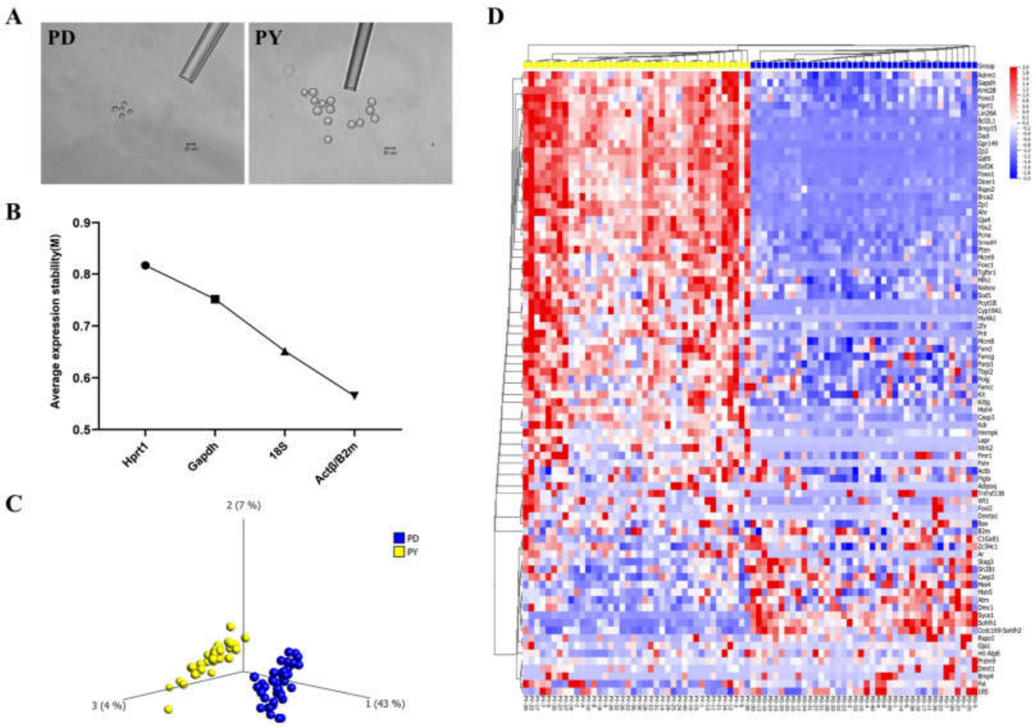

Supplementary Table S1. Gene information of custom TaqMan array cards

Genes	Assay IDs	Genes	Assay IDs
Ahr	Mm00478932_m1	*Dazl*	Mm01273546_m1
Bax	Mm00432051_m1	*Adipoq*	Mm00456425_m1
Casp2	Mm01160323_m1	*Zp3*	Mm00442176_m1
Eef2K	Mm00432996_m1	*Zfx*	Mm03053842_s1
Figla	Mm00488823_m1	*Ybx2*	Mm00497476_m1
Foxo3	Mm01185722_m1	*Stra8*	Mm00486473_m1
Fshr	Mm00442819_m1	*Dmrt1*	Mm00443809_m1
Gdf9	Mm00433565_m1	*Atm*	Mm01177457_m1
Kit	Mm00445212_m1	*Adrm1*	Mm00727402_s1
Kitlg	Mm00442972_m1	*Vgf*	Mm01204485_s1
Lep	Mm00434759_m1	*Pcyt1B*	Mm00616920_m1
Lin28A	Mm00524077_m1	*Fancc*	Mm00514846_m1
Mei4	Mm00613706_m1	*Tgfbr1*	Mm00436964_m1
Parp1	Mm01321084_m1	*Sh2B1*	Mm01137783_m1
Pcna	Mm00448100_g1	*Ptger2*	Mm00436051_m1
Smad4	Mm03023996_m1	*Ntrk2*	Mm00435422_m1
Spo11	Mm00488876_m1	*Gpr149*	Mm00805216_m1
Tbpl2	Mm01337558_m1	*C1Galt1*	Mm01167001_m1
Vip	Mm00660234_m1	*Robo2*	Mm00620713_m1
Zc3Hc1	Mm01168068_m1	*Sod1*	Mm01344233_g1
Casp3	Mm01195085_m1	*Prdm9*	Mm01279317_m1
Ccdc169-Sohlh2	Mm00512314_m1	*Msh4*	Mm01320240_m1
Cxcr5	Mm00432086_m1	*Gja4*	Mm00433610_s1
Cyp19A1	Mm00484049_m1	*Fst*	Mm00514982_m1
Gja1	Mm01179639_s1	*Fancl*	Mm00840321_m1
Ms4A1	Mm00545909_m1	*Fancg*	Mm00474063_m1
Ngf	Mm00443039_m1	*Bmp4*	Mm00432087_m1
Nkx2-3	Mm01199403_m1	*Bcl2L1*	Mm00437783_m1
Prlr	Mm04336676_m1	*Dicer1*	Mm00521722_m1
Rspo1	Mm00507077_m1	*Dmrta1*	Mm00558696_m1
Rspo2	Mm00555790_m1	*Foxc1*	Mm01962704_s1

Rxrg	Mm00436411_m1	*Hnrnpk*	Mm04409831_s1
Tnf	Mm00443258_m1	*Kdr*	Mm01222421_m1
Tnfrsf13B	Mm00840182_m1	*Kmt2B*	Mm01175393_g1
18S	Hs99999901_s1	*Lepr*	Mm00440181_m1
Gapdh	Mm99999915_g1	*Zp1*	Mm00494367_m1
Hprt1	Mm00446968_m1	*Brca2*	Mm01218747_m1
Actb	Mm00607939_s1	*Foxo1*	Mm00490671_m1
B2m	Mm00437762_m1	*Mcm8*	Mm00508536_m1
Ar	Mm00442688_m1	*Mcm9*	Mm01179858_m1
Bmp15	Mm00437797_m1	*Msh5*	Mm00488974_m1
Dmc1	Mm00494490_m1	*mt-Atp6*	Mm03649417_g1
Fmr1	Mm01339582_m1	*Nog*	Mm01297833_s1
Foxl2	Mm00843544_s1	*Polg*	Mm00450527_m1
Lhcgr	Mm00442931_m1	*Pten*	Mm00477208_m1
Nobox	Mm00453743_m1	*Stag3*	Mm00450460_m1
Sohlh1	Mm01338424_g1	*Wt1*	Mm01337048_m1
Mlh1	Mm00503449_m1	*Syce1*	Mm01279053_m1

Supplementary Table S2 primer sequences

Name	Accession number	Forward primer (5'→3')<br>Reverse primer (5'→3')
Actb	NM_007393.5	GGCTGTATTCCCCTCCATCG
		CCAGTTGGTAACAATGCCATGT
Pten	NM_008960.2	TGGATTCGACTTAGACTTGACCT
		GCGGTGTCATAATGTCTCTCAG
Foxo3	NM_001376967.1	CTGGGGGAACCTGTCCTATG
		TCATTCTGAACGCGCATGAAG
Nobox	NM_130869.3	ATGGAACCTACGGAGAAGCTC
		CTCAGAGGTCTTCGACAGTGG
Amh	NM_007445.3	CCACACCTCTCTCCACTGGTA
		GGCACAAAGGTTCAGGGGG
Gdf9	NM_008110.2	TCTTAGTAGCCTTAGCTCTCAGG
		TGTCAGTCCCATCTACAGGCA
Casp3	NM_001284409.1	GAGCTTGGAACGGTACGCTA
		CCGTACCAGAGCGAGATGAC
Bcl2	NM_009741.5	GAACTGGGGGAGGATTGTGG
		GCATGCTGGGGCCATATAGT
Il-6	NM_001314054.1	CCAAGAGGTGAGTGCTTCCC
		CTGTTGTTCAGACTCTCTCCCT
Ikbkb	NM_001159774.1	GTAGCAAAGTCCGAGGTCCC
		GTCTAGAGTCGTGAAGCTTCTGT
